# Supplementary material for: Identification of a neurocircuit underlying regulation of feeding by stress-related emotional responses
Source: Nat Commun. 2019 Aug 1;10:3446. doi: 10.1038/s41467-019-11399-z (PMC6671997; doi:10.1038/s41467-019-11399-z)
Supplement: Supplementary file 1 — Supplementary Information [file 41467_2019_11399_MOESM1_ESM.pdf]

Supplementary Figures

By Xu et al., in the manuscript entitled **"Identification of a Neurocircuit Underlying Regulation of Feeding by Stress-related Emotional Responses"** in *Nature Communications*.

Total 10 supplementary figures are included.

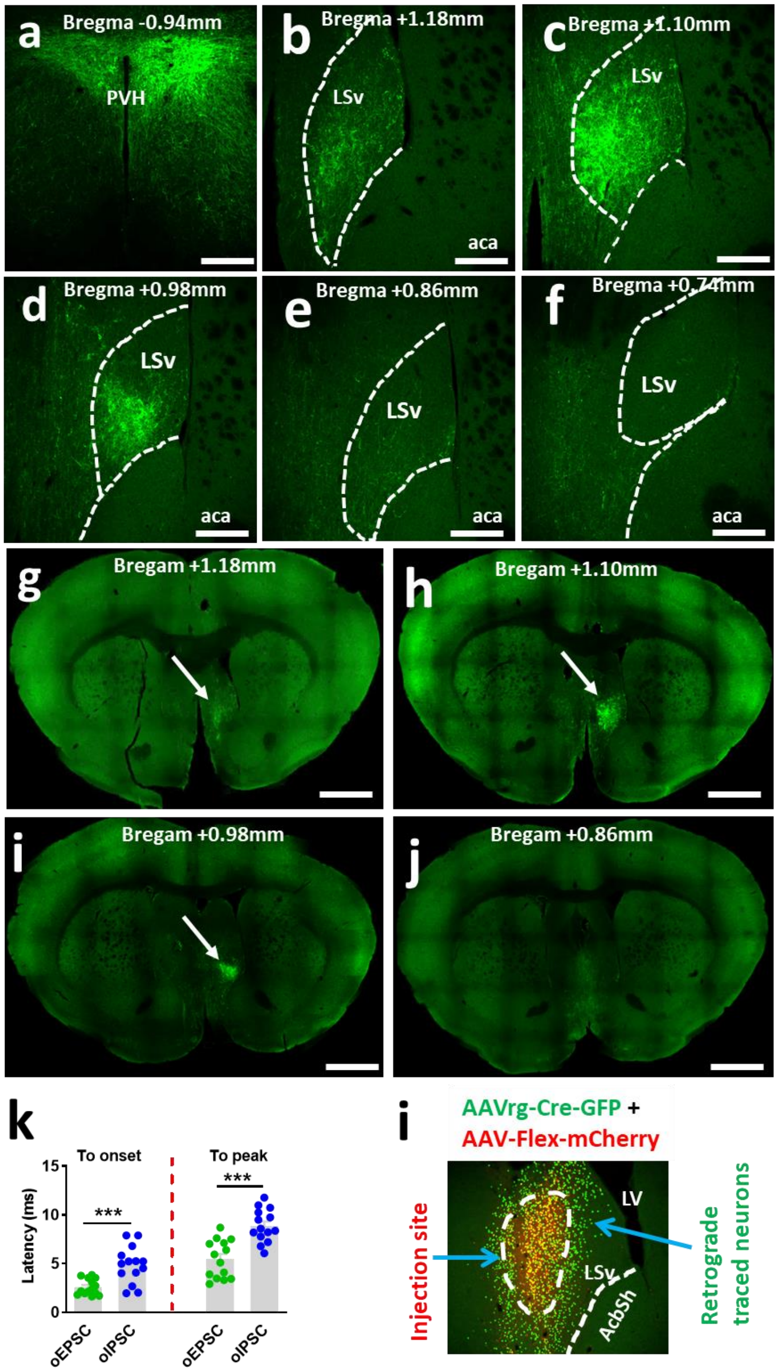

Supplementary Figure 1. Projection of PVH to the ventral part of lateral septum (LSv). Sim1-Cre mice with PVH specific delivery of AAV-Flex-ChR2-eGFP (a) and brain sections containing GFP-positive fibers across the lateral septum (LS) were shown from rostral to caudal directions in a high magnification (b-f) and a low magnification (g-j). (k) Neurons in the ventral part of lateral septum (LSv) were obtained for whole-cell patch recording for laser induced postsynaptic currents (oEPSCs and oIPSCs). Comparison of latency from the onset of laser illumination to the appearance of the onset and peak between the recorded IPSCs and EPSCs. (i) Coinjections of retro-AAV vector AAVrg-GFO-GFP and AAV-Flex-mCherry to LSv showing that AAVrg-Cre-GFP vecotrs in the hit neurons (mCherry and GFP colocalized neurons) could retrograde label neurons (GFP-only neurons) within the LSv. ***p<0.001, n=14, student's t tests. Arrows indicating only GFP-positive fibers observed in the LSv. Scale bar: 100µM in a-f and 500µM in g-j. aca: anterior commissure anterior part. LSv: ventral part of lateral septum; LV: lateral ventricle.

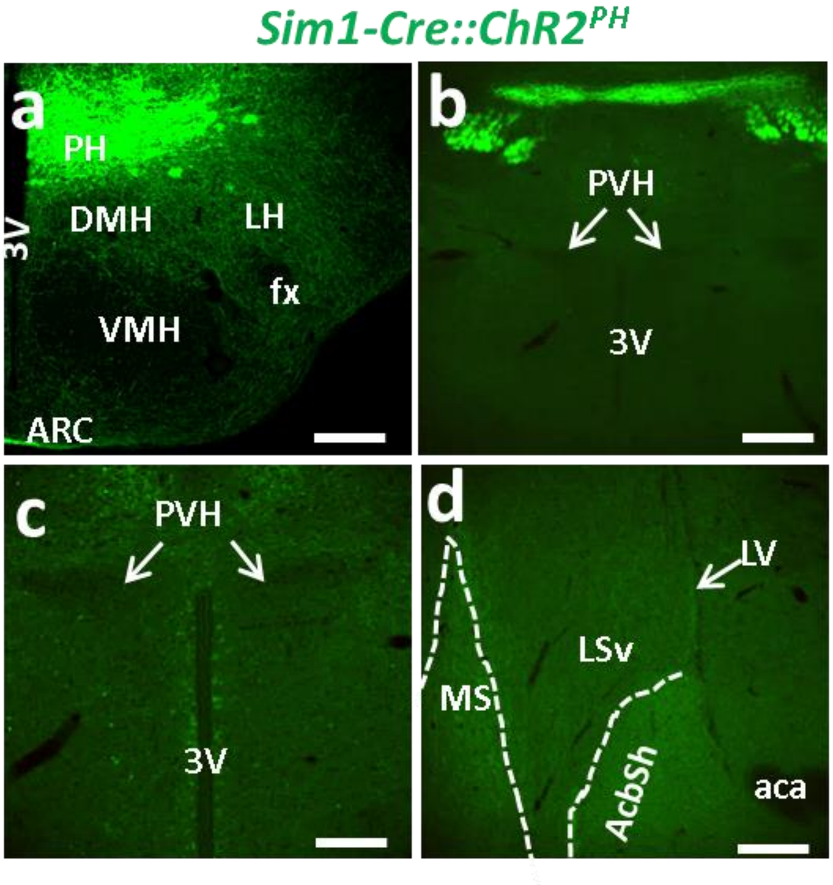

Supplementary Figure 2. Posterior hypothalamus (PH) does not project to LSv. Sim1-Cre reporter mice with PH specific delivery of AAV-Flex-ChR2-eGFP vectors showing specific expression of GFP in the PH (a), but not in the PVH at both rostral (b) and caudal levels (c). There were no GFP-expressing fibers found in the LSv (d). Scale bar: 100μM in a-d. PH: posterior hypothalamus; DMH: dorsomedial hypothalamus; VMH: ventromedial hypothalamus; LH: lateral hypothalamus; fx: fornix; PVH: paraventricular hypothalamus; 3V: the third ventricle; SCN: suprachiasmatic nucleus; LSv: ventral part of lateral septum; LV: lateral ventricle; AcbSh: accumbens shell; anterior commissure; MS: medial septum.

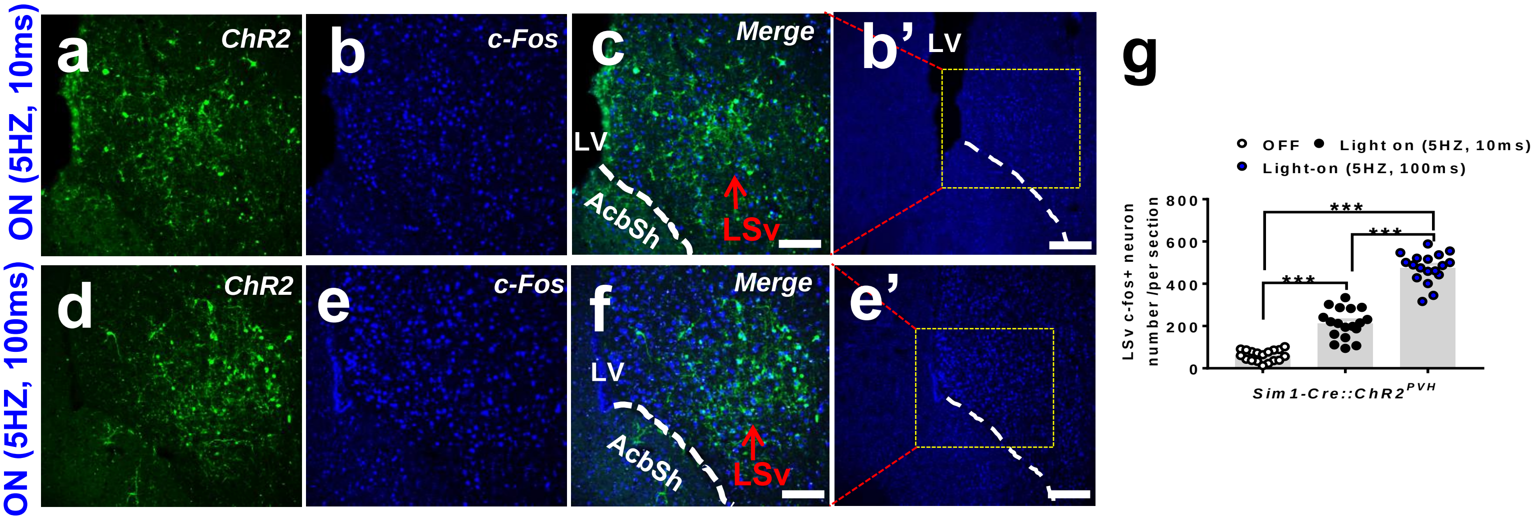

Supplementary Figure 3. Photostimulation of PVH→LSv fibers induces a scalable effect on LSv neuron activation. Sim1-Cre mice with specific delivery of AAV-Flex-ChR2-eGFP to the PVH and GFP-expressing fibers were observed in the LSv (a and d). Photostimulation of PVH^{Sim1}→LSv fibers caused c-Fos expression in LSv neurons (b and e). The photostimulation used either 5Hz, 5mW/mm^2, 10ms (short duration, a, b and c) or 5Hz, 5mW/mm^2 (long duration, d, e and f). Panels c and f are merged ones of a and b, and d and e, respectively. Dashed boxes in b' and e' represents the higher magnified area of a-c and d-f respectively. g: quantitative analysis of comparison in number of c-Fos positive neurons in LSv between the short and long duration stimulations. Scale bar: 50μM in a-f; 100 μMin b' and e'. One way ANOVA, ***p<0.001.

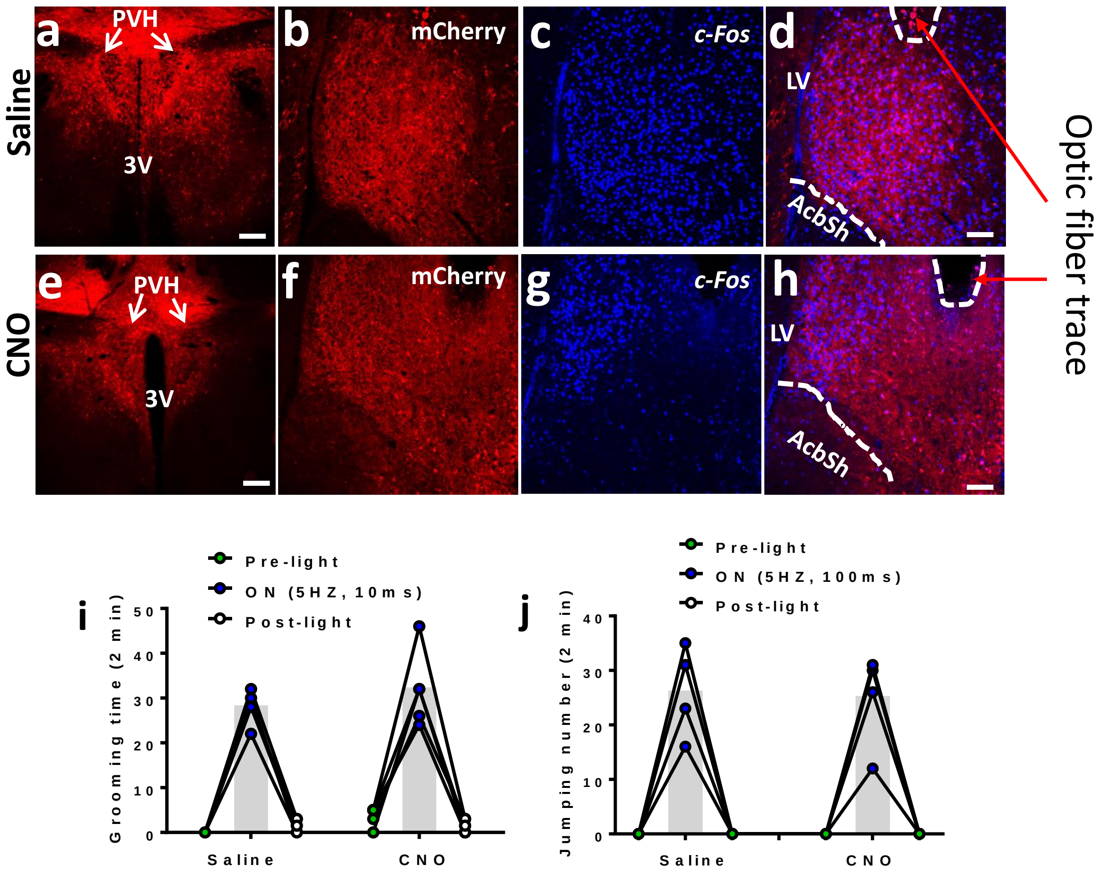

Supplementary Figure 4. Expression of AAV-fas-ChR2-mCherry and RTPP tests. *Vgat-Cre* mice with PVH specific delivery of AAV-fas-ChR2-mCherry vectors and LSv specific delivery of AAV-Flex-Gi-DREADD-mCherry vectors showed expression of mCherry in the PVH (a and e, arrows), Gi-DREADD expression in the LSv (b and f). AAV-fas-ChR2-mCherrry is expressed in all non-Vgat expressing neurons in the PVH. Laser stimulation of local LSv PVH→LSv fibers produced c-Fos in numerous number of LSv neurons with saline injection (c), which was obviously reduced by CNO injection (g). Panels d and h showed merged pictures of b and c, and f and g, respectively. (i and j) CNO administration alone had no effect on self-grooming (i) or jumping (j) elicited by photoactivation of PVH → LSv fibers in mice ChR2 expression in PVH Sim1 neurons but no DREADD expression in the LSv. White dashed circles outlined optic fiber tract. Scale bar=100µM.

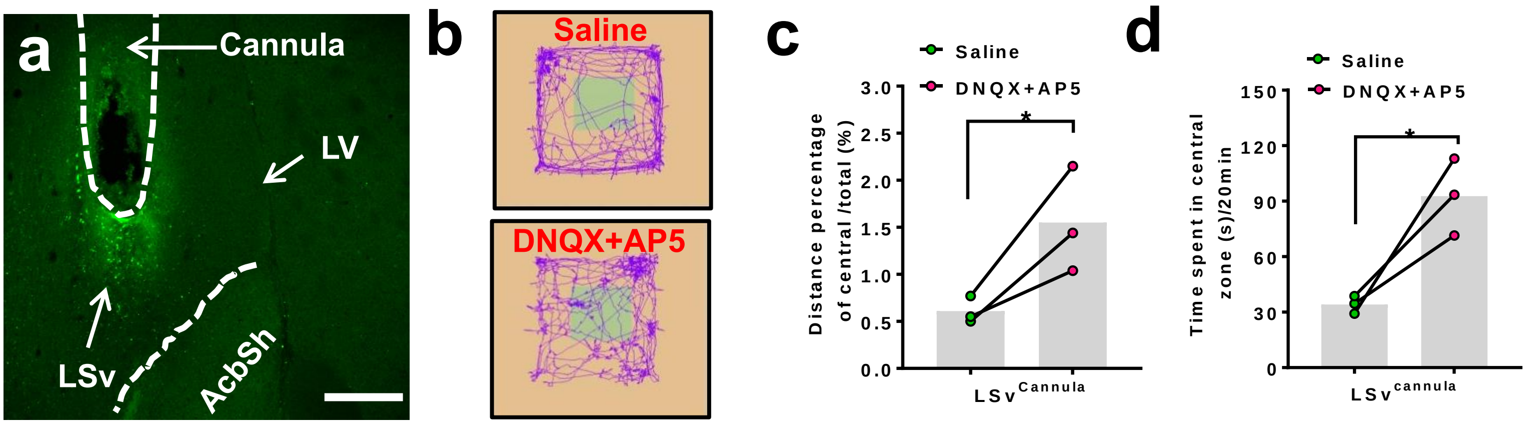

Supplementary Figure 5. Inactivation of glutamate receptors in LSv reduces anxiety. In Sim1-Cre mice with specific delivery of AAV-Flex-ChR2-eGFP to the PVH and GFP-expressing fibers were observed in the LSv, in which dual cannula were implanted targeting LSv for both photostimulation and drug infusion (a). Inactivation of glutamate receptors in LSv with infusion of DNQX+AP5 to the LSv through the cannula caused increased time spent in the center of open field assays, as shown in a representative activity trace (b) and statistical analysis from 3 animals (c and d). LV: lateral ventricle; LSv: the ventral part of lateral septum. Acbsh: accumben shell; scale bar=100μM. *p<0.05, Student's t test.

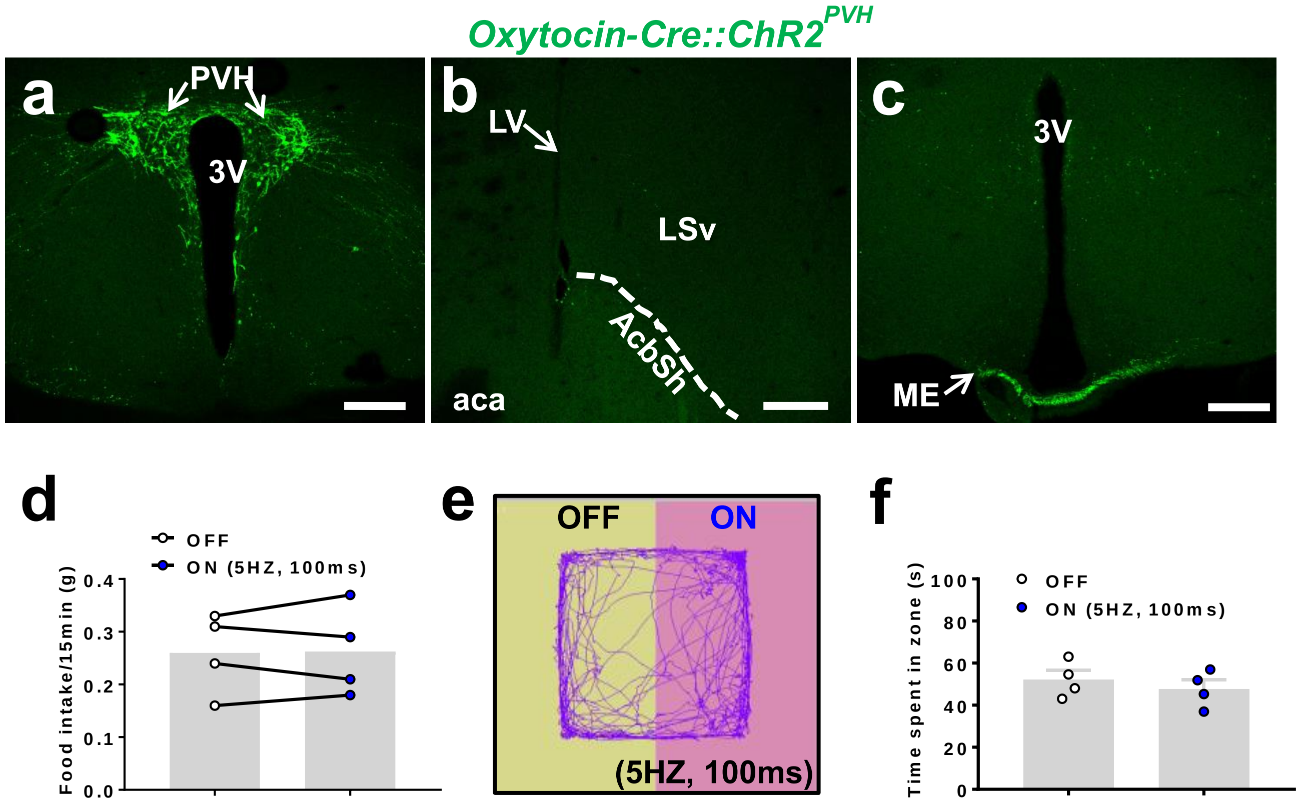

Supplementary Figure 6. PVH oxytocin neurons do not project to LSv. Oxytocin-Cre male mice with specific delivery of AAV-Flex-ChR2-eGFP to the PVH and 4 weeks after, GFP expression was observed in the PVH (a), but not in the LSv (b). Some GFP-expressing fibers were observed in the median eminence (c), presumably those that project to the posterior pituitary. (d-f) Oxytocin-Cre mice with specific delivery of AAV-Flex-ChR2-eGFP to the PVH and optic fibers implantation targeting LSv, feeding (d) and real time place preference (RTPP) tests were conducted with photpstimulation (5Hz, 100ms) (e-f). Photostimulation of $PVH^{oxytocin}$-LSv fibers has no effects on either fast-refeeding (d) or place preference (e-f). Overnight fasting mice were used to test fast-refeeding. PVH: paraventricular hypothalamus; 3V: the third ventricle; LSv: ventral part of laternal septum; LV: lateral ventricle; aca: anterior commissure area; ME: median eminence. Scale bar=100µM. ns: not significant, student's t tests. N=4 each.

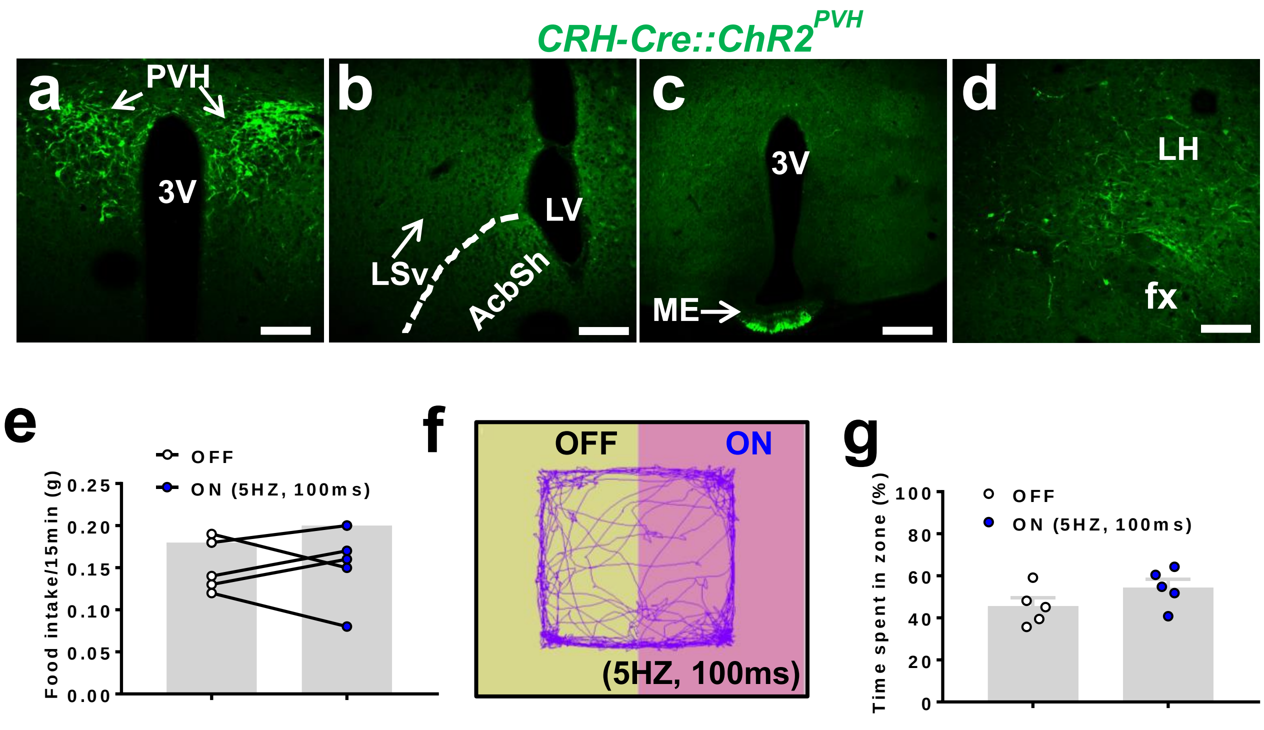

Supplementary Figure 7. PVH CRH neurons contribute insignificantly to PVH→LSv action. CRH-Cre mice with specific delivery of AAV-Flex-ChR2-eGFP to the PVH and 4 weeks after, GFP expression was observed in the PVH (a), but not in the LSv (b). Some GFP-expressing fibers were observed in the median eminence, presumably reflecting those that project to median eminence (c) and LH (d). Photostimulation of PVH^{CRH}-LSv fibers has no effects on either fast-refeeding (e) or place preference (f-g). Overnight fasting mice were used to test fast-refeeding. Interestingly, we observed some GFP-expressing fibers in the LSv 3 months after AAV vector delivery to the PVH (data not shown); however, photostimulation of PVH^{CRH}-LSv fibers 3 months after vector delivery failed to produce any difference in behavior (data not shown). The reason underlying this time-dependent appearance of GFP-expressing from PVH CRH neurons to LSv is unclear. PVH: paraventricular hypothalamus; 3V: the third ventricle; Acbsh: accumben shell; LSv: ventral part of laternal septum; LV: lateral ventricle; ME: median eminence; LH: lateral hypothalamus; fx: fonix. Scale bar=100μM. ns: not significant, student's t tests. N=5 each.

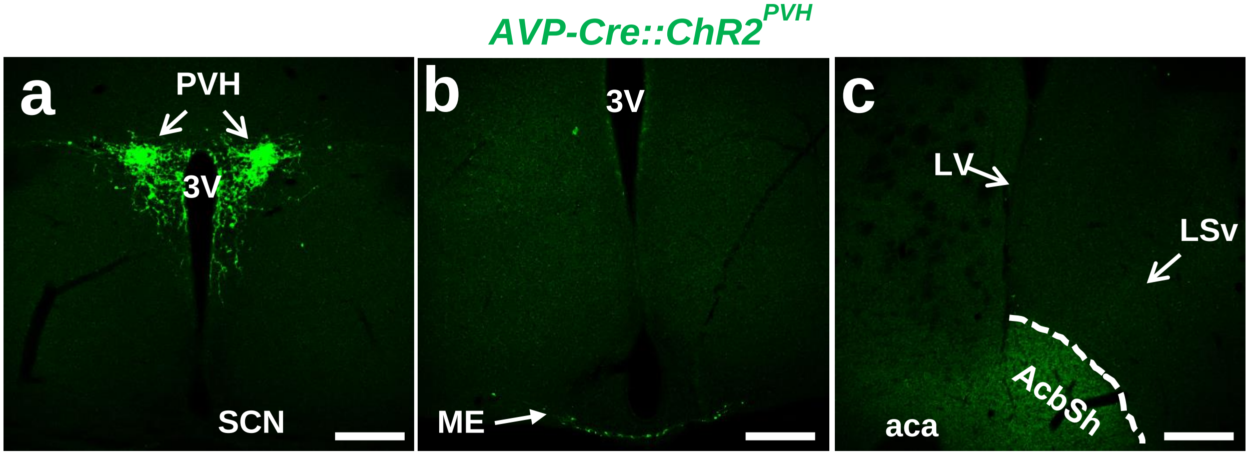

Supplementary Figure 8. PVH AVP neurons do not project to LSv. Four weeks after delivery of AAV-Flex-ChR2-eGFP vectors to the PVH of AVP-Cre mice, expression of eGFP was observed in the PVH (a) and GFP-positive fibers were observed in the median eminence area (b, arrow), likely reflecting projections to the posterior pituitary, but no GFP-positive fibers were found in the LSv (c, arrow). PVH: paraventricular hypothalamus; 3V: the third ventricle; SCN: suprachiasmatic nucleus; ME: median eminence; LV: lateral ventricle; LSv: ventral part of lateral septum; aca: anterior part of the anterior commissure. Scale bar=100µM.

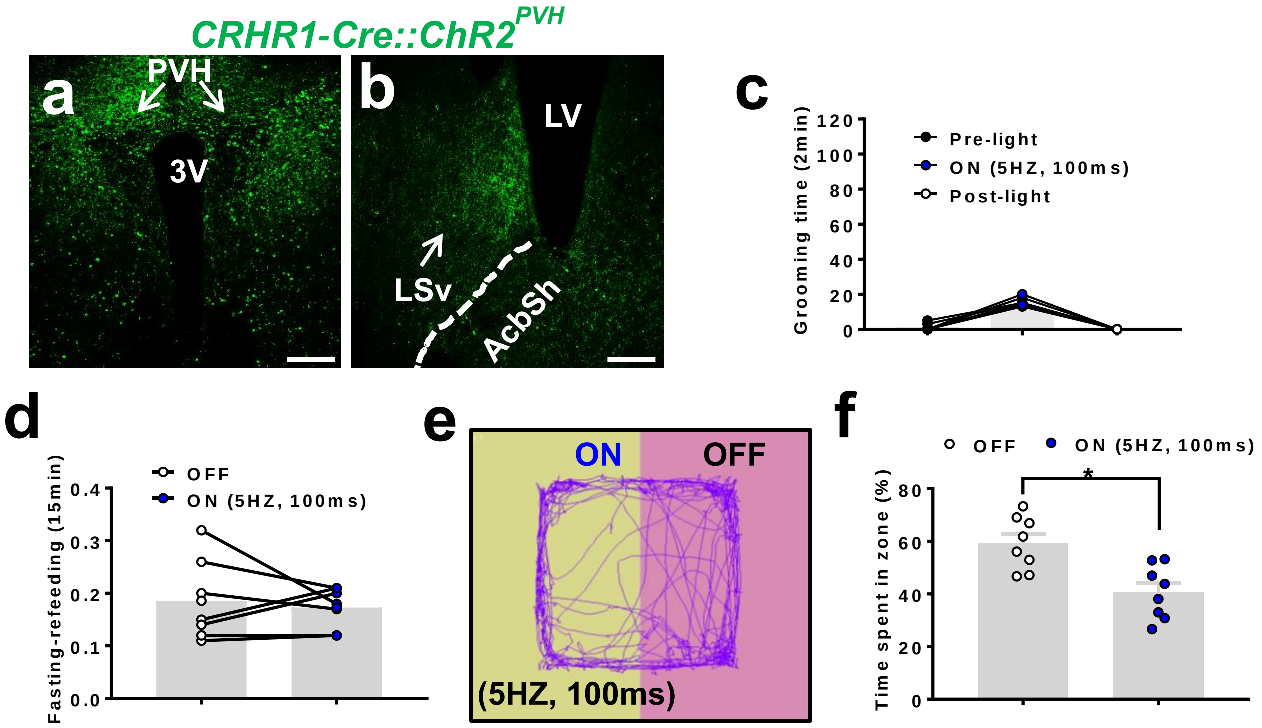

Supplementary Figure 9. CRHR1 (CRH receptor 1) neurons contribute partly to the PVH→LSv projection. (a-b) AAV-Flex-ChR2-eGFP vectors were delivered to the PVH of CRHR1-Cre mice, and neurons that express eGFP were found within the PVH as well outside the PVH boundary, reflecting the expression pattern of CRHR1-Cre (a) and eGFP-expressing fibers were also found in the LSv (b). Photostimulation (5Hz, 5mW, 100ms) of the eGFP-expressing fibers elicited mild self-grooming behavor (c), but no changes in refeeding after 12hr fasting (d), and a small increase in aversion during RTPP tests (e and f). *$p<0.05$, paired student's t tests. Scale bar = 100 µm.

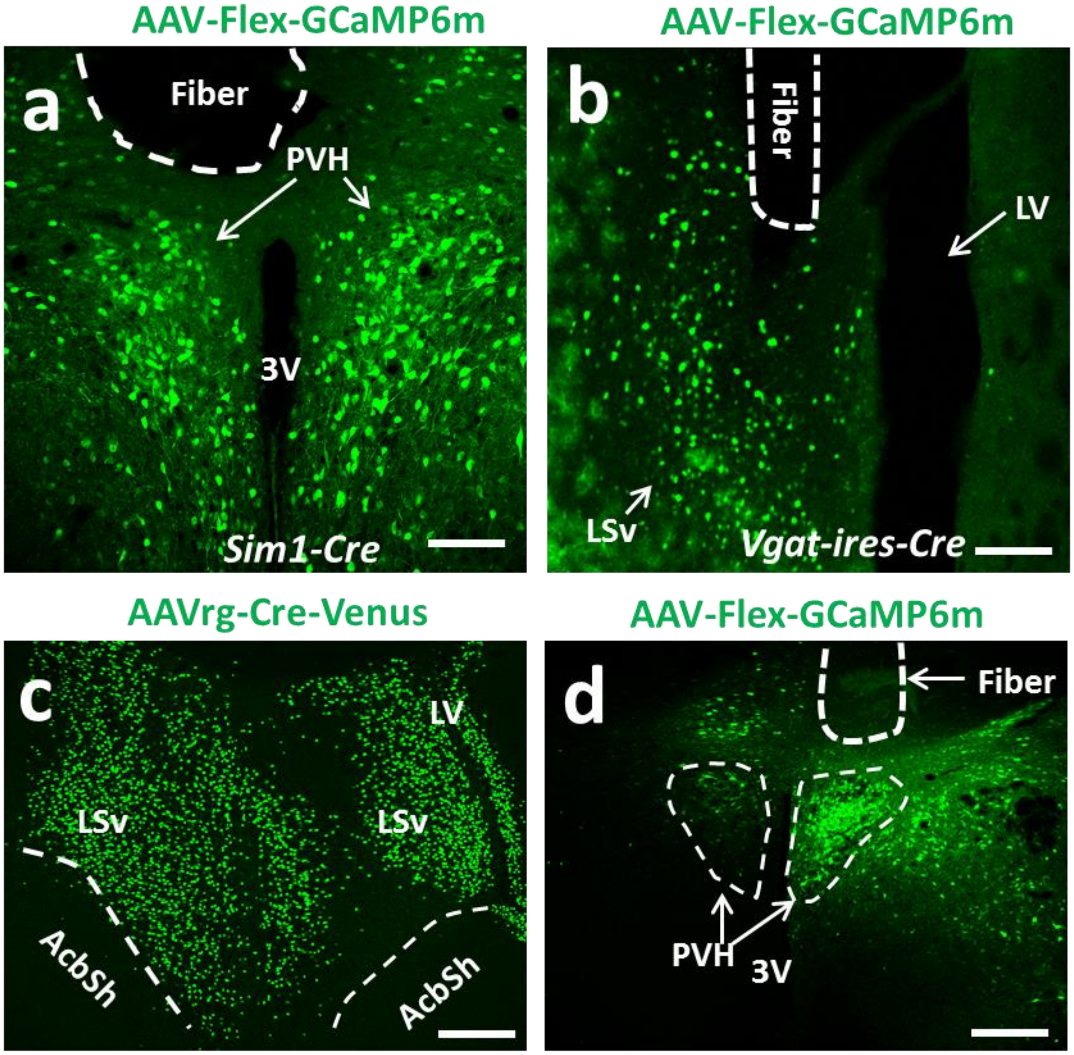

Supplementary Figure 10. Expression of GCaMP6m in the PVH and LSv. Four weeks after delivery of AAV-Flex-GCaMP6m to the PVH of Sim1-Cre mice and LSv of Vgat-Cre mice, brain sections were obtained and representative expression in the PVH (a, arrows) and LSv (b) was presented. (c-d) Wild type mice with delivery of retrograde AAV vectors AAVrg-Cre-Venus to bilateral LSv (c) received AAV-FLEX-GCaMP6m to the PVH (d), showing abundant GCaMP6m expression in Cre-retrograde traced PVH neurons. 3V: the third ventricle; LV; lateral ventricle; LSv: ventral part of lateral septum; Acbsh: accumben shell;. Scale bar = 50 µm in a and b, 100 µm in c and d.
