# Supplementary material for: Identification of a neurocircuit underlying regulation of feeding by stress-related emotional responses
Source: Nat Commun. 2019 Aug 1;10:3446. doi: 10.1038/s41467-019-11399-z (PMC6671997; doi:10.1038/s41467-019-11399-z)
Supplement: Supplementary file 4 — Description of Additional Supplementary Files [file 41467_2019_11399_MOESM4_ESM.pdf]

Description of Additional Supplementary Files

File Name: Supplementary Movie 1
Description: Short pulse photostimulation of PVH→LSv projections induces self-grooming behavior.

File Name: Supplementary Movie 2
Description: Long pulse photostimulation of PVH→LSv projections induces frantic escape jumping behavior.

File Name: Supplementary Movie 3
Description: Photostimulation of PVH→LSv projections reduces aggressive behavior.

File Name: Supplementary Movie 4
Description: Photostimulation of PVH→LSv produces rapid and reversible feeding inhibition.

File Name: Supplementary Movie 5
Description: Short pulse LSv GABAergic neurons produces rapid and reversible feeding inhibition.

File Name: Supplementary Movie 6
Description: Blockage of LSv glutamate receptors induces feeding in well-fed mice.
